# Association of Benign Prostatic Hyperplasia With Multimorbidity Among Older Adults: Insights From the Longitudinal Ageing Study in India (LASI), First Wave

**DOI:** 10.7759/cureus.50608

**Published:** 2023-12-15

**Authors:** Pritam Halder, Yukti Bhandari, Aritrik Das, Anshul Mamgai

**Affiliations:** 1 Department of Community Medicine and School of Public Health, Postgraduate Institute of Medical Education and Research, Chandigarh, IND

**Keywords:** lasi, multimorbidity, morbidity, bph, benign prostatic hyperplasia

## Abstract

Introduction

Population ageing is expected to be accompanied by an increase in multi-morbidity, i.e. the co-occurrence of multiple chronic conditions simultaneously. Benign prostatic hyperplasia (BPH) is a non-malignant disease prevalent in ageing men. Both BPH and multi-morbidity are known to have a significant impact on quality of life. The objective of this study was to determine the association between BPH and multimorbidity among older adults and the elderly population in India.

Methods

This is an analytical cross-sectional study involving secondary data from the nationally representative Longitudinal Ageing Study in India (LASI) Wave I 2017-18. Multivariable logistic regression analysis was conducted to study the association between BPH and multimorbidity while accounting for other associated factors.

Results

Compared to those having no co-morbidities, the odds of having BPH increased with the increasing number of co-morbidities. Those with at least two co-morbidities were twice as likely (aOR=2.19; 95%CI 1.78-2.72), and those with at least four co-morbidities were almost six times as likely (aOR=5.78; 95%CI 2-16.72) to have BPH as compared to those with no co-morbidities. The association was stronger among males >60 years.

Conclusion

Self-reported benign prostatic hyperplasia was found to be strongly associated with multi-morbidity. The need of the hour is the inclusion of BPH within the framework of a national health programme. Health technology assessment of high-risk screening strategies for BPH may be conducted among patients with multimorbidity. Research into the impact on the quality of life of those affected by both BPH and multimorbidity will help highlight this as a priority problem for decision-makers.

## Introduction

Benign prostatic hyperplasia (BPH) is the non-malignant enlargement of the prostate gland. It refers to stromal and glandular epithelial hyperplasia that occurs in the periurethral transition zone of the prostate. It clinically manifests with irritative (frequency, urgency, nocturia) and obstructive symptoms (straining to initiate urination, hesitancy, a weak and interrupted urine stream, and a sensation of incomplete bladder emptying) [1]. It is a disease prevalent in older men, with a previously reported age-specific prevalence of 8%, 50%, and 80% in the age groups of 30-39, 50-59, and 80-89 years, respectively [2]. Analysis of data from the Global Burden of Disease 2019 found that the absolute burden peaked in men aged 65-69 years and the age-specific prevalence was highest in men aged 75-79 years [3].

Despite the increase in the absolute burden of cases, the global age-standardised prevalence (2380 per 1,00,000 population in 2019, -0.77% change from 2000) and disability-adjusted life years (DALY) (48.9 per 1,00,000 population in 2019) attributed to the disease remained largely unchanged between 2000 and 2019. India has reported a high burden of 3480 (2640-4470) cases per 1,00,000 population. This reflects a 0.78% decrease in age-standardised prevalence from 2000, but a 90.9% increase in the number of cases from 95,50,000 in 2000 to 1,82,00,000 in 2019 [3].

Previous literature has reported several factors associated with benign prostatic hyperplasia including genetics, high dairy, poultry, protein, and energy intake, physical inactivity, sedentary lifestyle, and obesity [4,5]. Moderate alcohol intake and smoking appear to have an inverse association with BPH. Access to health care, level of education, socioeconomic status, and presence of other chronic diseases and co-morbidities were significant predictors for health-related quality of life among older BPH patients [5]. Co-morbidities including dyslipidemia, cardiovascular disease, diabetes mellitus, and metabolic syndrome among others have been found to be associated with increased occurrence of BPH [6-8].

Multimorbidity is a term used to describe the coexistence of multiple health conditions in an individual, which has been gaining increasing prominence [9]. It is an emerging global public health challenge with advancing medical science, increasing life expectancy, ageing populations, epidemiological transition and the rising prevalence of chronic conditions. Data from a previous nationally representative survey among adults aged 15-49 years have found the prevalence of multimorbidity in India to be 7.2%, with prevalence peaking up to 16% in some states [10]. In older populations, the prevalence ranges from 10.1-25.8% [11].

Temporal trends over the years suggest that population growth and ageing have a major impact on the prevalence and DALYs associated with benign prostatic hyperplasia at the global level [3]. This may ring especially true for India in the near future, where recent estimates suggest that 10.5% of the population was older than 60 years in 2022, and is expected to double to 20.8% by 2050 [12]. This also means that the proportions of those with chronic diseases and multi-morbidities are expected to rise in the coming years. The extent of the role that the burden of multiple conditions might play in the development of BPH needs to be studied further along with the different factors associated with the same among the older population in India. With this in mind, we analysed data from a nationally representative survey to determine the association between BPH and multimorbidity among older adults (45-59 years) and elderly (>60 years) population in India.

## Materials and methods

This was an analytical cross-sectional study comprising a secondary analysis of the Longitudinal Ageing Study in India (LASI) first-wave data from 35 Indian states and union territories (UTs), except Sikkim, approved by the Indian Council of Medical Research [11]. As a secondary data analysis of LASI, ethical approval was not needed for the current study.

LASI was a nationally representative longitudinal survey that collected detailed information on the psychological, social, economic, and health aspects of ageing in India. It was created to provide comprehensive and globally comparable survey data on ageing in the Indian context. The National Institute on Ageing, the Government of India's Ministry of Health and Family Welfare, and the United Nations Population Fund provided funding for the study. The University of Southern California, the International Institute for Population Sciences, and the Harvard T.H. Chan School of Public Health worked together on executing the survey methodology [11].

Wave 1 of the LASI covered a sample of 72,250 individuals aged 45 and above and their spouses. The study, which is one of the biggest of its kind in the world and the first of its kind in India, evaluated the scientific evidence in the context of variables like demographics, household economic status, chronic health conditions, symptom-based health conditions, functional health, mental health (cognition and depression), biomarkers, healthcare utilisation, family and social networks, social welfare programmes, employment, retirement, satisfaction, and life expectations.

LASI Wave 1 adopted a three-stage sampling design in rural areas and a four-stage sampling design in urban areas. In each state/UT, the first stage involved the selection of primary sampling units (PSUs), that is, sub-districts (tehsils/talukas), and the second stage involved the selection of villages in rural areas and wards in urban areas in the selected PSUs. In rural areas, households were selected from selected villages in the third stage. However, sampling in urban areas involved an additional stage. Specifically, in the third stage, one census enumeration block (CEB) was randomly selected in each urban area. In the fourth stage, households were selected from this CEB. To ensure the inclusion of an adequate number of those aged over 65 years, an oversampling of households with at least one member over 65 years of age was done [11].

Outcome variable

Self-reported BPH was assessed by questioning, “Have you ever been diagnosed with any of the following urogenital conditions or diseases?”, with options including BPH.

Explanatory variables

For this study, data was extracted only of male participants aged 45 years and above. The explanatory variables of interest were any co-morbidity and multimorbidity. The following chronic health conditions were considered while accounting for multimorbidity: hypertension, diabetes, chronic lung diseases (e.g. chronic obstructive pulmonary disease, asthma, chronic bronchitis, other chronic lung problems), chronic heart disease (e.g. congestive heart failure, myocardial infarction, heart attack, other chronic heart diseases), dyslipidaemia (high cholesterol), and thyroid disorders. To assess the presence of chronic health diseases or conditions, the interviewer asked the question “Has any health professional ever diagnosed you with the following chronic conditions or diseases?”. This question required a dichotomous (No/Yes) response. Participants having at least two chronic health conditions were described as multimorbidity.

Covariates

Following variables were taken as covariates to assess adjusted association: Age-group (years 45-59, >= 60), minimum educational qualification (illiterate, less than primary, primary completed, middle completed, secondary school, higher secondary, and diploma/ graduate), residence (rural, urban), marital status (unmarried, married/live-in, widow/separated/divorced), monthly per capita expenditure (MPCE) (poorest, poorer, middle, richer, richest) quintile, health insurance (no, yes), occupation (unemployed, professional and semi-professional: legislators and senior officials, professionals, technicians and associate professionals, clerical and skilled: clerks, service workers and shopkeepers, skilled agriculture and fishery workers, craft and related trade worker, plant and machine operator, unskilled), physical activity (everyday, once per week, one to three times per week, once per month, never), BMI <18.5, 18.5-22.9, 23-24.9, 25-29.9, >30, self-rated health (excellent, very good, good, fair, poor,), tobacco usage (no, yes), and alcohol consumption (no, yes).

**Figure 1:**
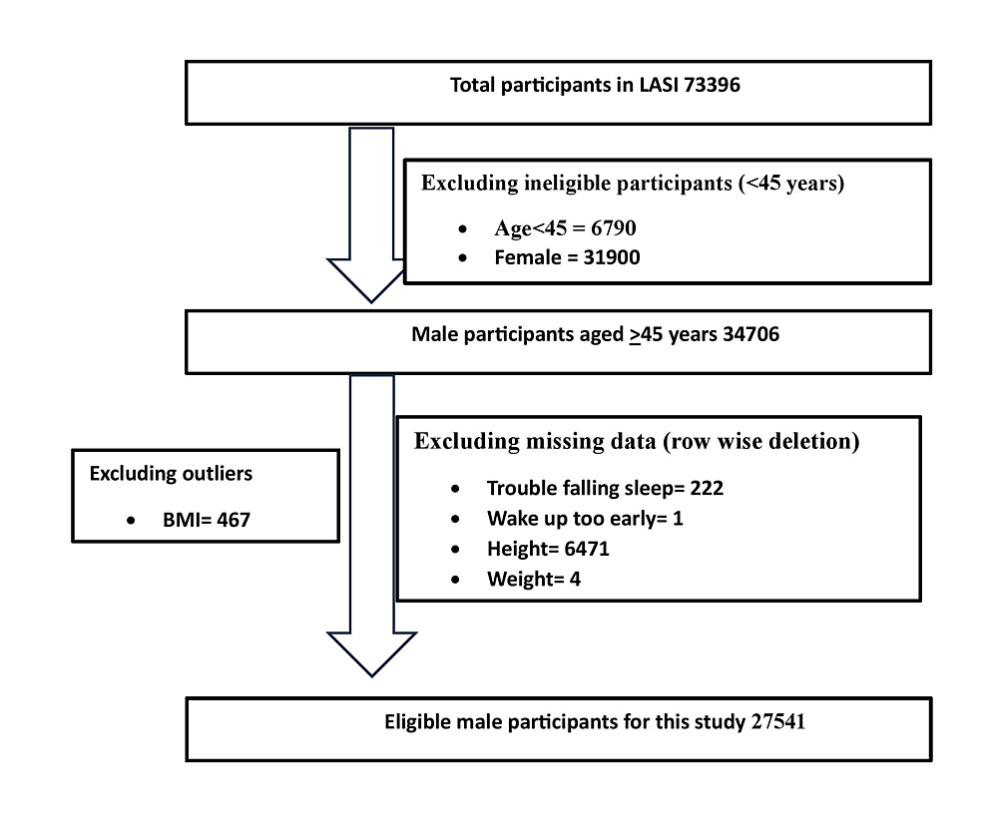
Flowchart showing participant selection process in this study LASI: Longitudinal Aging Study in India

After adjusting missing data by row-wise deletion and excluding BMI outliers, we included participants who had documented their BPH status. The details are provided in Figure 1. Thus, this study included information from 27,541 participants.

Statistical analysis

Data was analysed with Stata Statistical Software: Release 17 (StataCorp LLC, College Station, Texas, United States). Characteristics of participants were described as mean (standard deviation) for continuous variables and frequencies and percentage for categorical variables (age group, minimum education, MPCE quintile, occupation, BMI). Univariate logistic regression was conducted between the outcome variable and each explanatory variable. To avoid multicollinearity among explanatory variables, a variance inflation factor (VIF) was applied. VIF > 5 indicates a high correlation between a given explanatory variable and other explanatory variables in the model, which might create problems with the regression analysis. Variable with VIF>5 (e.g. marital status) was excluded from the final association. P-values <0.05 were considered statistically significant. Variables with univariate p-value of <0.2 were taken for further multivariable logistic regression. The association was calculated in the overall population and was further categorised as per age group.

## Results

A total of 27,541 study participants were included in the analysis. Their mean ± SD age (years) was calculated to be 60.06 ± 10.55 (45-59 years age group = 51.39 ± 4.27, ≥60 years age group = 68.89 ± 7.21).

Among the study population, BPH was self-reported by 410 males (1.49%), of which 144 (1.04%) were between 45-59 years old and 266 (1.95%) were over the age of 60 years. On univariate analysis, BPH in the overall population (≥45 years) was significantly associated with age group, education status, MPCE quintile, having health insurance, occupation, and physical activity. Among the population aged ≥ 60 years, BPH was significantly associated with education status, place of residence, MPCE quintile, having health insurance, occupation, and BMI categories (Table 1).

**Table 1:** Various characteristics of Indian male population aged >45 years, 45-59 years and >60 years BPH: Benign Prostatic Hyperplasia; MPCE: Monthly Per-capita Consumer Expenditure

Variable	Overall population (>45 years)			Older adults (45-59 years)			Elderly (>60 years)		
	Total (N=27,541), N (%)	BPH (N=410; 1.49%), N (%)	p-value	Total (N=13,900), N (%)	BPH (N=144; 1.04%), N (%)	p-value	Total (N=13,637), N (%)	BPH (N=266; 1.95), N (%)	p-value
Age (years)									
45-59	13,903 (50.48%)	144 (1.04%)	<0.001	-	-	-	-	-	-
>60	13,638 (49.52%)	266 (1.95%)		-	-	-	-	-	-
Education (minimum)									
Illiterate	8,637 (31.36)	111 (1.29)	<0.001	3,675 (26.43)	34 (0.93)	0.315	4,962 (36.38)	77 (1.55)	<0.001
Less than primary	3,785 (13.74)	42 (1.11)		1,750 (12.59)	12 (0.69)		2,035 (14.92)	30 (1.47)	
Primary completed	4,329 (15.72)	57 (1.32)		2,243 (16.13)	25 (1.11)		2,086 (15.30)	32 (1.53)	
Middle completed	3,502 (12.12)	45 (1.28)		2,088 (15.02)	20 (0.96)		1,414 (10.37)	25 (1.77)	
Secondary school	3,388 (12.30)	70 (2.07)		1,844 (13.26)	25 (1.36)		1,544 (11.32)	45 (2.91)	
Higher Secondary	1683 (6.11)	37 (2.20)		1055 (7.59)	16 (1.52)		628 (4.60)	21 (3.34)	
Diploma/Graduate	2217 (8.05)	48 (2.17)		1248 (8.98)	12 (0.96)		969 (7.11)	36 (3.72)	
Residence									
Rural	18,334 (66.57)	260 (1.42)	0.173	9,074 (65.27)	103 (1.14)	0.112	9,260 (32.73)	157 (1.70)	0.002
Urban	9,207 (33.43)	150 (1.63)		4,819 (34.73)	41 (0.85)		4,378 (32.10)	109 (2.49)	
Marital Status									
Unmarried	398 (1.45)	5 (1.26)	0.288	246 (1.77)	3 (1.22)	0.949	152 (1.11)	2 (1.32)	0.788
Married/live-in	24,247 (88.40)	354 (1.45)		13,043 (93.81)	135 (1.04)		11,304 (82.89)	219 (1.94)	
Widow/separated/divorced	2,796 (10.15)	51 (1.82)		614 (4.42)	6 (0.98)		2,182 (16.00)	45 (2.06)	
MPCE quintile									
Poorest	5,440 (19.75)	55 (1.01)	0.014	2,711 (19.50)	19 (0.70)	0.264	2,729 (20.01)	36 (1.32)	0.023
Poorer	5,556 (20.17)	80 (1.44)		2,763 (19.87)	29 (1.05)		2,793 (20.48)	51 (1.83)	
Middle	5,570 (20.22)	88 (1.58)		2,781 (20.00)	32 (1.15)		2,789 (20.45)	56 (2.01)	
Richer	5,590 (20.30)	93 (1.66)		2,870 (20.64)	37 (1.29)		2,720 (19.94)	56 (2.06)	
Richest	5,385 (19.55)	94 (1.75)		2,778 (19.98)	27 (0.97)		2,607 (19.12)	67 (2.57)	
Health insurance									
No	26,484 (96.16)	383 (1.45)	0.004	13,348 (96.01)	136 (1.02)	0.336	13,136 (96.32)	247 (1.88)	0.002
Yes	1,057 (3.84)	27 (2.55)		555 (3.99)	8 (1.44)		3.68 (3.68)	19 (3.78)	
Occupation									
Unemployed	8,485 (30.81)	171 (2.02)	<0.001	1,457 (10.48)	10 (0.69)	0.111	7,028 (51.53)	161 (2.29)	0.023
Professional and semi-professional	1,003 (3.64)	13 (1.30)		802 (5.77)	9 (1.12)		201 (1.47)	4 (1.99)	
Clerical and skilled	9,898 (35.94)	133 (1.34)		6,141 (44.17)	77 (1.25)		3,757 (27.55)	56 (1.49)	
unskilled	8,155 (29.61)	93 (1.14)		5,503 (39.58)	48 (0.87)		2,652 (19.45)	45 (1.70)	
Physical activity									
Everyday	8,745 (31.75)	106 (1.21)	0.024	5,449 (39.19)	61 (1.12)	0.054	3,296 (24.17)	45 (1.37)	0.055
More than once / week	2,392 (8.69)	40 (1.67)		1,443 (10.38)	18 (1.25)		949 (6.96)	22 (2.32)	
Once / week	1,172 (4.26)	15 (1.28)		659 (4.74)	6 (0.91)		513 (3.76)	9 (1.75)	
1-3 times /month	1,503 (5.46)	33 (2.20)		819 (5.89)	15 (1.83)		684 (5.02)	18 (2.63)	
Never	13,729 (49.85)	216 (1.57)		5,533 (39.80)	44 (0.80)		8,196 (60.10)	172 (2.10)	
BMI Categories									
<18.5	5,282 (19.18)	61 (1.16)	0.122	2,047 (14.72)	20 (0.98)	0.594	3,235 (23.72)	41 (1.27)	0.018
18.5-22.9	11,389 (41.35)	180 (1.58)		5,664 (40.74)	53 (0.94)		5,725 (41.98)	127 (2.22)	
23-24.9	4,425 (16.07)	69 (1.56)		2,444 (17.58)	26 (1.06)		1,981 (14.53)	43 (2.17)	
25-29.9	5,493 (19.94)	80 (1.46)		3,195 (22.98)	36 (1.13)		2,298 (16.85)	44 (1.91)	
>30	952 (3.46)	20 (2.10)		553 (3.98)	9 (1.63)		399 (2.93)	11 (2.76)	
Ever used tobacco									
No	12,131 (44.05)	171 (1.41)	0.337	6,180 (44.45)	58 (0.94)	0.312	5,951 (43.64)	113 (1.90)	0.702
Yes	15,410 (55.95)	239 (1.55)		7,723 (55.55)	86 (1.11)		7,687 (56.36)	153 (1.99)	
Ever consumed alcohol									
No	18,198 (66.08)	260 (1.43)	0.251	8,832 (63.53)	87 (0.99)	0.435	9,366 (68.68)	173 (1.85)	0.197
Yes	9,343 (33.92)	150 (1.61)		5,071 (36.47)	87 (1.12)		4,272 (31.32)	93 (2.18)	

Table 2 depicts the prevalence of different co-morbidities and multi-morbidity in the study population and its association with BPH. Two-thirds (66.06%) of the population had some form of morbidity, while multimorbidity with at least two co-morbidities was prevalent in 34.65%. In the elderly age group, multimorbidity with at least two co-morbidities and at least three co-morbidities was prevalent in 43.95% and 10.32% of the population, respectively. BPH was significantly associated with having hypertension, diabetes, chronic heart disease, dyslipidemia, and thyroid disorders in the study population. It was also associated with multi-morbidity having at least two, three, and four co-morbidities. Among participants aged 45-59 years, BPH was significantly associated with hypertension, chronic heart disease, and thyroid disease. It was also associated with co-morbidity and multimorbidity with at least two co-morbidities . Among participants aged ≥60 years, BPH was significantly associated with having hypertension, diabetes, dyslipidemia, and thyroid disease. It was also associated with having any co-morbidity and multimorbidity. 

**Table 2:** Prevalence of comorbidity and multimorbidity among Indian male population aged >45 years, 45-59 years, and >60 years BPH: Benign Prostatic Hyperplasia

Variable	Overall population (>45 years)			Older adults (45-59 years)			Elderly (>60 years)		
	Total (N=27,541), N (%)	BPH (N=410), N (%)	p-value	Total (N=13,903), N (%)	BPH (N=144), N (%)	p-value	Total (N=13,637), N (%)	BPH (N=266), N (%)	p-value
Hypertension	6,706 (24.35)	149 (2.22)	<0.001	2,560 (18.41)	46 (1.80)	<0.001	4,146 (30.40)	103 (2.48)	0.003
Diabetes	3,570 (12.96)	81 (2.27)	<0.001	1,399 (10.06)	19 (1.36)	0.210	2,171 (15.92)	62 (2.86)	0.001
Chronic lung disease	1,694 (6.15)	34 (2.01)	0.069	582 (4.19)	5 (0.86)	0.667	1,112 (8.15)	29 (2.61)	0.098
Chronic heart disease	1,151 (4.18)	31 (2.69)	0.001	355 (2.55)	8 (2.25)	0.022	796 (5.84)	23 (2.89)	0.48
Dyslipidemia	858 (3.12)	29 (3.38)	<0.001	380 (2.73)	6 (1.58)	0.289	478 (3.50)	23 (4.81)	<0.001
Thyroid disease	329 (1.19)	19 (5.78)	<0.001	155 (1.11)	7 (4.52)	<0.001	174 (1.28)	12 (6.90)	<0.001
At least one morbidity	18,193 (66.06)	331 (1.82)	<0.001	8,055 (57.94)	102 (1.27)	0.002	10,138 (74.34)	229 (2.26)	<0.001
Multimorbidity (at least 2)	9,542 (34.65)	234 (2.45)	<0.001	3,548 (25.52)	60 (1.69)	<0.001	5,994 (43.95)	174 (2.90)	<0.001
Multimorbidity (at least 3)	2,018 (7.33)	59 (2.93)	<0.001	611 (4.39)	9 (1.47)	0.275	1,407 (10.32)	50 (3.56)	<0.001
Multimorbidity (at least 4)	62 (0.23)	4 (6.45)	0.001	14 (0.10)	0 (0.00)	0.702	48 (0.35)	4 (8.33)	0.001

Bivariate and multivariable logistic regression analysis was conducted to explore the association between BPH and multi-morbidity in the study population. Model 1 was adjusted for age, while model 2 was adjusted for all the factors found to be associated with BPH with p-value < 0.2 in Table 1.

Compared to those having no morbidity, the odd of having BPH increased with thr presence of any other morbidity and with an increasing number of co-morbidities included in the multi-morbidity (Table 3). Those with at least two co-morbidities, were twice as likely (aOR = 2.19; 95%CI 1.78-2.72), and those with at least four co-morbidities were almost six times as likely (aOR = 5.78; 95%CI 2-16.72) to have BPH as compared to those with no co-morbidities. 

**Table 3:** Univariate and multivariable logistic regression of BPH and comorbidity/multimorbidity Model 1= adjusted for age; Model 2= adjusted for age, education, residence, MPCE quintile, health insurance, occupation, physical activity, BMI category, ever used tobacco, ever consumed alcohol Classification accuracy= 98.51% Model 2 Pseudo R2= 0.0384 MPCE: Monthly Per Capita Expenditure; BPH: Benign Prostatic Hyperplasia

Characteristics	Benign Prostatic Hyperplasia					
	Univariate		Multivariable			
	Crude odds ratio (95% CI)	p-value	Adjusted odds ratio (95% CI) Model-1	p-value	Adjusted odds ratio (95% CI) Model-2	p-value
No morbidity	Reference	-	Reference	-	Reference	-
At least one co-morbidity	2.17 (1.7-2.78)	<0.001	1.97 (1.54-2.52)	<0.001	1.78 (1.37-2.31)	<0.001
Multimorbidity (at least 2)	2.55 (2.09-3.10)	<0.001	2.32 (1.90-2.84)	<0.001	2.19 (1.78-2.72)	<0.001
Multimorbidity (at least 3)	3.53 (2.51-4.97)	<0.001	3.01 (2.13-4.26)	<0.001	2.63 (1.82-3.81)	<0.001
Multimorbidity (at least 4)	6.10 (2.87-22.82)	<0.001	6.61 (2.33-18.72)	<0.001	5.78 (2.00-16.72)	0.001

Similar results were seen when subgroup analysis was conducted among older adults and the elderly. Table 4 depicts the results of the bivariate and multivariable logistic regression analysis to explore the strength of the association of BPH with the presence of any co-morbidity and multi-morbidity in the study population. Among both the 45-59 and >60 years age groups, those with multimorbidity with at least two or more co-morbidities were more than twice as likely to have BPH as compared to those with no morbidity.

**Table 4:** Association of BPH and comorbidity/multimorbidity according to age group Adjusted for education, residence, MPCE quintile, health insurance, occupation, physical activity, BMI category, ever used tobacco, ever consumed alcohol Classification accuracy= 98.05% Pseudo R2= 0.0393 MPCE: Monthly Per Capita Expenditure; BPH: Benign Prostatic Hyperplasia

Age group	Benign Prostatic Hyperplasia			
	Univariate		Multivariable	
	Crude odds ratio (95%CI)	p-value	Adjusted odds ratio (95%CI)	p-value
45-59 years (N= 13903)				
No morbidity	Reference	-	Reference	-
At least one co-morbidity	1.77 (1.24-2.54)	0.002	1.80 (1.23-2.62)	0.002
Multimorbidity (at least 2)	2.10 (1.51-2.94)	<0.001	2.17 (1.54-3.11)	<0.001
Multimorbidity (at least 3)	2.07 (1.00-4.27)	0.050	2.21 (1.05-4.70)	0.038
Multimorbidity (at least 4)	-	-	-	-
>60 years (N= 13638)				
No comorbidity	Reference	-	Reference	-
At least one comorbidity	2.16 (1.52-3.07)	<0.001	1.82 (1.27-2.62)	0.001
Multimorbidity (at least 2)	2.46 (1.90-3.17)	<0.001	2.19 (1.66-2.85)	<0.001
Multimorbidity (at least 3)	3.45 (2.25-5.30)	<0.001	2.69 (1.70-4.27)	0.004
Multimorbidity (at least 4)	6.10 (2.87-22.82)	<0.001	5.78 (2.00-16.72)	0.001

## Discussion

The projected increase in the population of older and elderly adults over the next few decades will be accompanied by an increase in the prevalence of chronic non-communicable diseases as well as benign prostatic hyperplasia [12]. The commonality of the risk factors behind several of these chronic diseases has heralded the co-occurrence of multiple conditions simultaneously, now described as multi-morbidity. The national representative LASI is a first-of-its-kind study on ageing in India, and the data collected from older and elderly adults allowed the exploration of the association between BPH and multi-morbidity in the Indian population [11].

The prevalence of BPH in the study population older than 45 years was 1.49%, which is lower compared to the Global Burden of Disease estimates of 3.4% (2.6-4.4%) [3]. The lower estimation may be due to the self-reported nature of the data on BPH in our study. Multimorbidity was reported by 34.65% of the study population. A meta-analysis of community-based studies found multimorbidity among 31% of the Asian male population [13]. In the population over the age of 60 years, multimorbidity was reported among 51% globally as compared to 43.95% in our analysis. The difference may be explained by the variations in definitions of multi-morbidity across studies.

Multimorbidity is known to have an impact on both physical and mental components of health-related quality of life [14]. Previous studies have also shown benign prostatic hyperplasia and its symptoms to be associated with poor quality of life [15]. Our analysis found that those with multi-morbidity were more likely to have BPH, with the odds increasing with the increase in the number of co-morbidities. This pattern of association was significant for both older adults from 45-59 years and the elderly over 60 years of age. BPH was found to be significantly associated with hypertension, diabetes, chronic lung disease, chronic heart disease, dyslipidemia, and thyroid disorders in our analysis. Previous studies from across the world have also found BPH to be significantly associated with several chronic diseases. Peng et al. in Taiwan found that male asthmatics were 1.4 times more likely to have BPH, though the odds decreased with increasing age [16]. Associations have also been reported between BPH and chronic kidney disease, chronic obstructive pulmonary disease, hypertension, thyroid disorders and metabolic syndrome [8,17-21]. Age and multiple co-morbidities are important predictors for requiring pharmacological treatment for alleviation of BPH symptoms [22]. Treatment for BPH has also been found to increase the risk for cardiovascular disease in patients, which only adds to the burden of co-morbidities [23]. Therefore, it is evident that the co-existence of BPH and multimorbidity is detrimental to the health and quality of life of patients and is not just an association of chance.

Our analysis also found that a higher proportion of those with secondary school education or higher, residing in urban areas, having health insurance, and belonging to richer and richest MPCE quintiles reported having BPH. Similar factors were identified in studies conducted in China [24], Korea [4], and the United States [25]. The association may be explained by better health-seeking behaviour among these sub-populations, coupled with wider access to appropriate screening and diagnostic services for BPH. Smoking and alcohol consumption have been reported to play a protective role in some studies [24,26] but no such association was found in our analysis. A lower proportion of those who engaged in daily physical activity reported BPH, which was corroborated by previous studies [24,4].

With the expected increase in the ageing population, accompanied by an increase in the number of co-morbidities due to the epidemiological transition in India, it is prudent to take steps to improve awareness regarding BPH and its symptoms among the general population. This should be coupled with the provision of affordable and accessible screening and diagnostic services for BPH so that there is an appropriate diagnosis and treatment for the condition. Targeted screening of high-risk groups, i.e. older and elderly adults with multi-morbidity may be considered for this purpose. All of this can be achieved by bringing benign prostatic hyperplasia under the ambit of the National Programme for Prevention and Control of Non-Communicable Diseases (earlier known as the National Programme for Prevention and Control of Cancer, Diabetes, Cardiovascular Diseases and Stroke) [26], or the National Programme for Health Care of the Elderly [27]. This will help prioritise a disease of ageing that has so far not found due prominence, and help improve the health-related quality of life amidst the ever-increasing burden of multimorbidity.

To the best of our knowledge, this secondary data analysis, conducted on data from a nationally representative sample is the first to explore to explore the association between BPH and multimorbidity among the Indian male population. However, there are certain limitations. The survey was cross-sectional in nature, it is only possible to establish associations without drawing any causal inference between variables. The outcome variable of benign prostatic hyperplasia is self-reported and not based on any objective diagnostic technique or criteria which may have resulted in misclassification of study participants. This is also the case with several of the reported co-morbidities that have been taken into account for multimorbidity. Since the data was already collected, it was not possible to gather information on health-related quality of life and similar indicators to provide deeper insight into the impact of BPH in those with multimorbidity.

## Conclusions

Self-reported BPH was found to be strongly associated with multi-morbidity, with the odds of having BPH increasing from twice as likely in individuals with at least two co-morbidities to almost six times as likely in individuals with at least four co-morbidities. The association was found to be stronger in those aged above 60 years. The inclusion of BPH within the framework of a national health programme is a policy decision that is the need of the hour. It would help to improve awareness of the disease and make screening and diagnostics more affordable and accessible.

Health technology assessment of high-risk screening strategies for BPH may be conducted among patients with multimorbidity. Further research into the possible pathophysiology and interactions of different chronic diseases in the development of BPH is warranted to better understand the epidemiology of the disease and design therapy to better treat multimorbidity as a whole. Research into the impact on the quality of life of those affected by both BPH and multimorbidity will also help highlight this as a priority problem for the decision-makers.
